# Mini-organs with big impact: Organoids in liver cancer studies

**DOI:** 10.32604/or.2023.029718

**Published:** 2023-07-21

**Authors:** MUHAMMAD BABAR KHAWAR, YAJUN WANG, ANEEQA MAJEED, ALI AFZAL, KABEER HANEEF, HAIBO SUN

**Affiliations:** 1 Institute of Translational Medicine, Medical College, Yangzhou University, Yangzhou, China; 2 Jiangsu Key Laboratory of Experimental and Translational Non-Coding RNA Research, Yangzhou, China; 3 Applied Molecular Biology and Biomedicine Lab, Department of Zoology, University of Narowal, Narowal, Pakistan; 4 Department of Oncology, Haian Hospital of Traditional Chinese Medicine, Haian, China; 5 Molecular Medicine and Cancer Therapeutics Lab, Department of Zoology, Faculty of Sciences Technology, University of Central Punjab, Lahore, Pakistan; 6 Chinese Institute for Brain Research (CIBR), Beijing, China

**Keywords:** Heterogeneity, Tumor, Organoid, Liver, Treatment, Therapy

## Abstract

Hepatocellular carcinoma, the most common primary liver cancer and a leading cause of death, is a difficult disease to treat due to its heterogeneous nature. Traditional models, such as 2D culture and patient-derived xenografts, have not proven effective. However, the development of 3D culture techniques, such as organoids, which can mimic the tumor microenvironment (TME) and preserve heterogeneity and pathophysiological properties of tumor cells, offers new opportunities for treatment and research. Organoids also have the potential for biomarker detection and personalized medication, as well as genome editing using CRISPR/Cas9 to study the behavior of certain genes and therapeutic interventions. This review explores to-the-date development of organoids with a focus on TME modeling in 3D organoid cultures. Further, it discusses gene editing using CRISPR/Cas9 in organoids, the challenges faced, and the prospects in the field of organoids.

## Introduction

Hepatocellular carcinoma (HCC) is one of the most common and most fatal diseases [1] making up about 80% of all liver cancer cases while the other type–the intrahepatic Cholangiocarcinoma (iCCA) contributes to 14.9% and 5% of cases, respectively [2]. Liver cancer is a leading cause of death, and the World Health Organization predicts that it will cause one million deaths per year by the end of 2030, becoming the third most lethal cancer [3]. As the burden of liver cancer on healthcare systems is increasing, it is essential to provide proper treatment for liver cancer [4]. In many cases, surgery is the last option and is feasible only during the early stages of HCC [5,6].

The current standard of care for liver cancer, such as chemotherapy and radiation, has limited efficacy and can cause severe side effects [7]. This has created a significant gap in the treatment of liver cancer, highlighting the need for new therapeutic strategies. One promising approach in liver cancer research is the use of organoids, which are miniaturized 3D structures that mimic the architecture and function of human organs. Organoids derived from liver cancer cells have been used to model the disease, study its molecular and cellular mechanisms, and test new drugs [8]. Organoids have several advantages over traditional cell culture models, as they better replicate the complexity and heterogeneity of tumors and allow for high-throughput drug screening [9]. Moreover, organoids can be derived from patient tumors, providing a personalized approach to cancer treatment [10,11]. Using organoid technology, researchers have now been successful in creating a variety of models, including those of the colon [12], liver [13], optic cup [14], pancreas [15], and lung [16].

Despite these advantages, there are still gaps in our understanding of organoids in liver cancer studies. Further research is needed to optimize the culture conditions and improve the reproducibility and scalability of organoids and the clinical translation of organoids as a therapeutic strategy for liver cancer requires rigorous validation in preclinical and clinical studies. This review covers the history and development of liver organoids, their advantages in modeling liver cancer and the tumor microenvironment (TME), gene editing using CRISPR/Cas9, and prospects, along with challenges in liver cancer research.

### History of the development of liver organoids to date

Liver organoid models have been developed from both healthy and diseased liver tissue using various techniques. The first successful organoid model was reported in 1985, where mouse intestinal stem cells were cultured in a 3D matrix. However, it took another 10 years before liver organoids were first reported in rats in 1995 [17,18]. These early studies were limited to rodent models only, but significant progress was made by Agrawal et al. in 2008 when human liver stem cells were used to create 3D structures [19]. The research has witnessed several significant advancements, including the use of iPSCs to create liver organoids, the establishment of protocols for long-term culture, and the development of co-culture systems with other liver cell types to better mimic the *in vivo* microenvironment [20]. However, despite these developments, there remain challenges in the use of liver organoids for HCC research, such as the need for standardized culture conditions, limited availability of patient samples, and the difficulty in recreating the liver tissue’s complexity. Nonetheless, the use of organoids in HCC research has provided valuable insights into the disease’s molecular mechanisms and has the potential to accelerate the discovery of new treatments and biomarkers [8].

Organoids have gained immense importance in the past decade, with a history of breakthroughs in liver organoid development being explained in Table 1. Among these breakthroughs, Ouchi et al. [21] generated a reproducible method to derive liver organoids from 11 different healthy and diseased pluripotent stem cell lines, which showed transcriptomic resemblance to *in vivo*-derived tissues. Under free fatty acid treatment, the organoids demonstrated key features of steatohepatitis, including steatosis, inflammation, and fibrosis phenotypes in a successive manner. Interestingly, the organoid-level biophysical readout with atomic force microscopy demonstrated that organoid stiffening reflects fibrosis severity [21]. These findings offer a new approach to studying personalized inflammation and fibrosis in humans, which can help in the discovery of effective treatments.

**TABLE 1:** Progressive breakthroughs in the development of liver organoids

Sr No.	Author	Year	Species	Cell source	Medium	Findings	Ref.
1	Landry et al.	1985	Rat	Hepatic cells	Williams’ medium E	Made 3D hepatocytes and bile duct-resembling cells	[22]
2	Huch et al.	2013	Mouse	Lgr5+liver stem cells	Wnt agonist RSPO1	Established long-term 3D mouse liver organoids	[23]
3	Takebe et al.	2013	Human	HUVECs, iPSCs, MSCs	Endothelial growth medium and hepatocyte culture medium	Reported functional liver organoids from iPSCs	[24]
4	Huch et al. 2015	2015	Human	Human adult bile-duct epithelial cells	Mouse liver culture medium (ERFHNic)	Established Human adult biliary epithelial-derived progenitor cells	[25]
5	Thomas et al.	2018	Mouse Human	Mouse and human hepatocytes	Hep-Medium	Cultured hepatocytes-derived organoids to obtain partial hepatectomy transcriptional profiles	[26]
6	Peng et al.	2018	Mouse	Primary Hepatocytes	3D Matrigel	TNF enhanced and allowed long-term culture for more than 6 months	[27]
7	Nuciforo et al.	2018	Human	Hepatocellular Carcinoma	Advanced DMEM/F-12	Cultured long-term organoid cultures of HCC with various etiologies and tumor stages	[28]
8	Ouchi et al.	2019	Human	Human PSC	mTeSR1 medium with matrigel coated plates	Precision medicine and modeling liver disease	[21]
9	Mun et al.	2019	Human	Human ESC Human iPSC	Matrigel-coated dishes and RPMI 1640	Developed novel human pluripotent stem cell-derived hepatocyte-like liver organoids	[29]
10	Ramli et al.	2020	Human	Human ESCs Human iPSCs	Dulbecco’s modified Eagle medium (DMEM)	Created a human cell-based hepatic organoid platform that may be utilized to mimic complicated liver diseases like NASH	[30]
11	Mo et al.	2022	Human	Liver metastasis tissues	Matrigel	Constructed a Biobank of 50 patient-derived organoids from a primary tumor and paired liver metastatic lesions	[31]

Note: ESC embryonic stem cells; HUVEC human umbilical vein endothelial cells; iPSC induced pluripotent stem cells; MSCs Mesenchymal stem cell; HCC hepatocellular carcinoma.

## Application of Organoids for Studying Specific Liver Cancers

Organoids are grown from primary tissue samples or stem cells under controlled conditions to form 3D structures that resemble the tissue of origin. To obtain liver organoids, liver tissue is dissociated into single cells and cultured under specific growth factors, extracellular matrix components, and culture media. Once established, organoids are characterized and used for various experimental assays, including the study of drug resistance, tumor initiation and progression, and identifying potential biomarkers for diagnosis and treatment of various diseases.

To understand the underlying mechanisms of specific liver cancers, such as HCC, CCA, and combined hepatocellular cholangiocarcinoma (CHC), there is a growing need for more sophisticated *in vitro* models that can recapitulate the *in vivo* environment. Regarding this, recent advances in organoid technology have provided a promising platform for studying the development and progression of liver cancer. For example, Turtoi et al. developed a new 3D model to study HCC by growing HepG2 cells on a scaffold made of hyaluronic acid (HA). They examined the response to the anti-tumor drug cisplatin by analyzing the cytotoxicity and apoptotic response of the cells. The scaffold allowed the cells to grow into larger clusters, exhibiting liver-like functions and expressing key hepatocyte-specific markers such as albumin, bile acids, and transaminases. Additionally, the scaffold increased the sensitivity of the hepatocytes to cisplatin, thereby enhancing its anti-tumor effect [32]. The cytotoxic and apoptotic response to cisplatin can potentially lead to more effective anti-tumor therapies. Li et al. [33] developed a bioprinter platform that incorporates both iCCA cells and stromal cells to investigate how stromal cells affect the behavior of CCA cells. This 3D bio-printed CCA model can mimic the TME more accurately and potentially serve as a clinically relevant platform for drug testing, offering an alternative to animal models [33]. The 3D bio-printed CCA model could be used to create a realistic simulation of the TME and can be a useful platform for preclinical research and drug testing, possibly replacing animal models.

Moreover, researchers created a new model for CCA using a combination of silk fibroin, HA, heparin sulfate, and gelatin that mimics tumor behavior better than 2D systems [34]. This new 3D model can yield cancer stem cells and offer a better representation of the microenvironment and drug sensitivity. Further investigations into the potential use of this model for drug screening and personalized medicine are warranted.

### Modeling liver cancer with organoids vs. monolayer cultures

**Figure 1:**
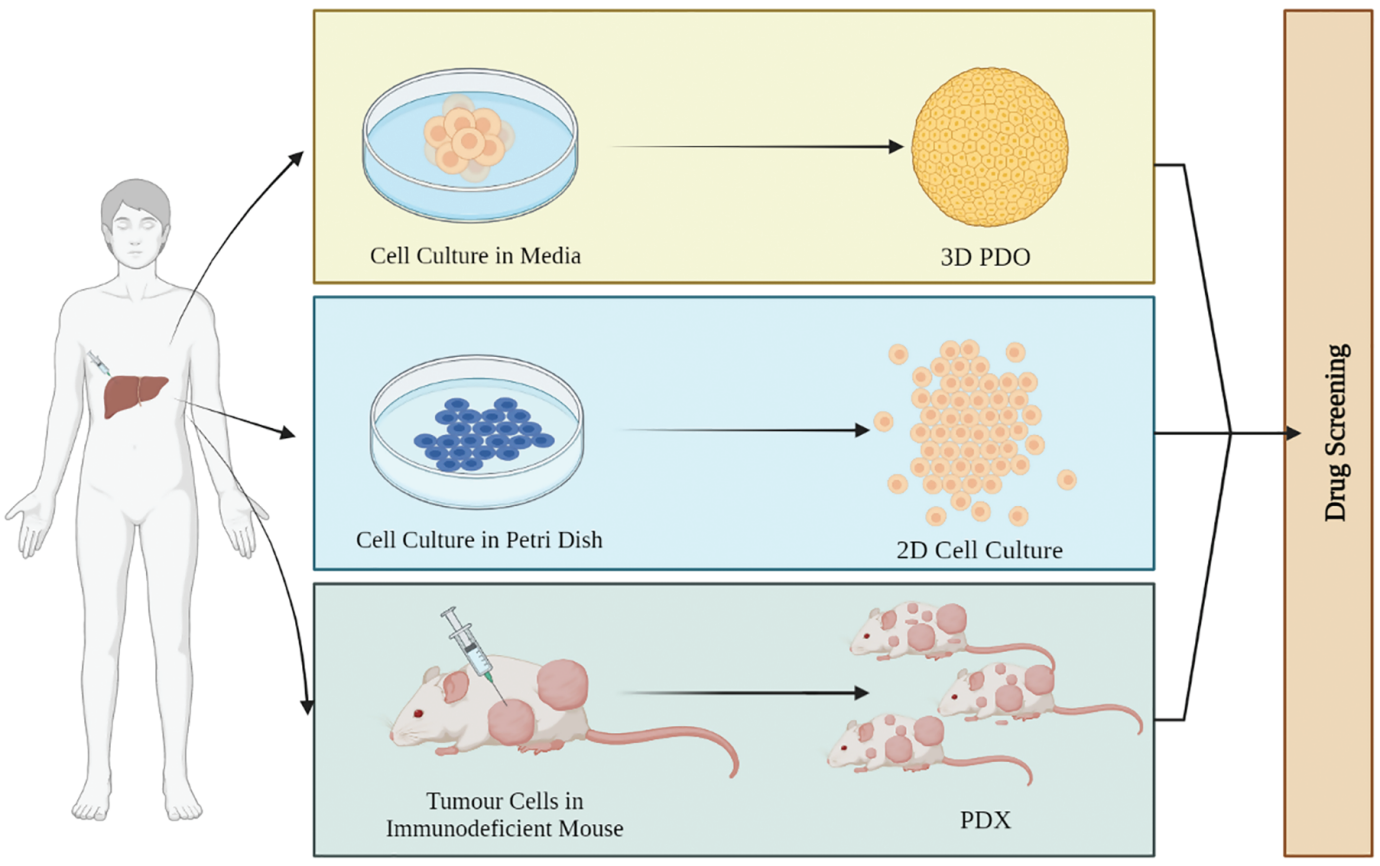
Schematic of different kinds of organoids. 3D patient-derived organoids (PDOs) obtained from the patient’s tumor mimic the TME of the patient with higher survival rates. 2D cell culture monolayer models lack stromal compartments and are unable to assist in the growth of normal cell lines used as controls. Patient-derived xenografts (PDXs) are useful for drug testing due to their dynamic framework and shape, providing valuable information on tumor tissue metabolism and drug efficacy.

The *in vitro* 3D structure is significantly more ideal for biomedical investigation because of its complex structure, microenvironment, and cellular pathways as compared to monolayer (Fig. 1). It also resembles the physiological environment of an organ *in vivo* more closely [35–37]. The 3D human liver organoids are more sensitive to drugs and less vulnerable to cytotoxicity as compared to monolayer cultures. For instance, the impact of drug-induced phospholipidosis (PL) was evaluated in both 2D and 3D cultures by Lee et al. Drug-induced PL was induced in 3D human liver organoids and monolayer HepG2 cultures. Organoids survive a concentration that was toxic to HepG2 monolayer cells during the 48-h incubation period. For instance, at the 20 µM dose of amiodarone and sertraline, both the 2D and 3D cultures died but the viability of organoids was much more compared to HepG2 [38]. A 2D monolayer culture cannot replicate 3D cellular interactions and non-cellular components that are present in the human body [36,37].

A 2D model was considered to be a vital model for drug screening and cancer research traditionally but it has limitations when it comes to recapitulating the heterogenicity of the tumor. Two-dimensional models have homogenous growth and proliferation and hence are not able to mimic the heterogeneous nature of the tumor [39,40]. On the other hand, organoids maintain their microenvironment and are better models for drug testing and determining drug response since tumor heterogeneity acts as a main challenge for any therapy [41,42]. van Tienderen et al. [43] recently reported the use of hydrogel which is derived from the extracellular matrix of the tumor to support the maintenance of tumor organoids and established a new pathway for its use as patient-specific medication [43]. Organoids are more significant than other models like spheroids and sandwich cultures when it comes to monitoring drug toxicity, metabolic activity, or cytoplasmic accumulations they are clinically more relevant.

### Tumor microenvironment modeling using 3D organoid cultures

The process of carcinogenesis, tumor growth, and propagation of cancerous cells involves complex interactions between cancerous cells and non-cancerous host elements that make up the TME [44]. The TME functions as a niche where the extracellular matrix can be soft or rigid depending on the type of tumor and is suspended with cancer-associated fibroblasts (CAFs), invading immune cells, blood, and lymphatic vascular networks [45]. The TME supports tumor growth and restricts therapeutic actions, while also suppressing immune response and maintaining a sufficient supply of oxygen and nutrients [46]. Due to the limitations of 2D models, 3D organoid cultures are increasingly being utilized to model TME and evaluate cancer therapeutics [42]. When compared to the 2D modeling approach, 3D organoid culture preserves the biomarker expression of primary cancer cells considerably better [47]. The extracellular vesicles secreted by 3D organoids also exhibit heterogeneous features, and the organoids share striking similarities with the tissue of the stem cell from which they are derived [48]. There are, in general, six different specialized microenvironments within the TME including the hypoxic niche, acidic niche, innervated niche, metabolic microenvironment, immunological microenvironment, and biomechanical microenvironment, each serving its purpose [49].

The diversity of a TME is not only influenced by gene mutations but also the tumor’s location, type, presence of lymph nodes and adipose tissue, and protein mutations (KRAS, EGFR, and PTEN) [50,51]. The development of specialized microenvironments through complex interactions between these components dictates the tumor’s immunogenicity and resistance to immunotherapy [51]. TME secretes growth factors that stimulate CAFs, which in turn support tumor cells by excreting ECM [52]. Many 3D cell-based models have been previously used like the 3D (hetero) spheroids model system, 3D-organoid tumor model system, microfluidics models, etc. However, each of these models comes with its own set of limitations. For example, the 3D (hetero) spheroids model systems lack reproducibility owing to the natural heterogeneity of the donor [53] and the infiltration of macrophages into the spheroid [54]. Microfluidic models offer precise control over experimental conditions, but they are expensive to produce and can be difficult to use for large-scale studies [55]. Organoids preserve the cellular pathophysiology of a tumor and tumor heterogeneity *in vitro* and are useful for the study of tumor-stroma interactions [51,56]. The existence of unique TME components facilitates molecular targeting for the inhibition of tumor development and metastasis [51]. It is not very simple to investigate the pathogenesis of any tumors using animal models since these studies take a lot of time and money and do not accurately replicate biological processes specific to humans [42]. Therefore, it is crucial to broadly assess tumor rates and conduct drug testing and research using the patient-derived xenografts (PDX) approach as tumor growth varies with patients [52].

The investigation of immune-oncology cannot be carried out using the PDX approach because PDXs are created in immunocompromised mice to avoid rejection, which means they lack components relevant to immunity [57,58]. Patient-derived organoids (PDO), an *in vitro* culture system, have a greater success rate than PDX since they are more affordable, less time-consuming to maintain, and require less effort. One of the best features of PDOs is that immune-oncology studies are possible [59]. Wang et al. experimented using sorafenib and Hedgehog signaling inhibitors for the treatment of HCC. They employ the PDO model to demonstrate the effectiveness of their therapy since PDO can preserve the histological features of the tumors from which they are derived. The success rate was shown to be 50 fold more than that of any other model had been used [60]. PDOs are a fundamental source for determining a patient’s tumor heterogeneity and cancer subtype and may be used to test for drugs that are efficient in killing tumorous cells without hurting healthy cells by combining organoids derived from healthy and tumor tissues [51].

### Comparison of the heterogeneity of organoids with the original tumor

Organoids preserve the 3D structure of organs as well as their histology, including their epithelial structure [61]. Organoids successfully mimic the distinctive surface markers of various tumors because they offer paired molecular profiling [62]. PDO has been used to culture many tumors type which were difficult to culture in the past like neuroendocrine tumors [63]. The same tumors can exhibit multiple levels of heterogeneity, which is significant from a clinical point of view since it requires various treatment developments and may also lead to resistance in the same patient. Organoid whole genome sequencing had been previously done to spot variations in karyotypes and analyze their progression over time [63]. Along with variations at a molecular level, organoids have morphological changes as well, for instance, Sharick et al. found that in the same kit well, some organoids have dense phenotypes while some are hollow [64,65]. Biobanks are important when it comes to investigating the heterogeneity of organoids and the original tumor as it facilitates effective drug testing, drug screening, and toxicity testing, facilitating specialized clinical treatment. The direct collection of tumors from patients is one way to create a tumor biobank, and the other is to modify organoids that are derived from healthy cell lines [66]. By surgically extracting liver tumor tissue from 8 individuals, Broutier et al. [67] created the first primary liver cancer organoids, which they referred to as tumoroids. They include subtypes of HCC, cryptogenic cirrhosis (CC), and CHC. Organoids can help identify possible prognostic biomarkers in neoplasms because of their heterogeneity. To investigate transcriptomic characteristics, Broutier et al. [67] compared primary liver cancer organoids with healthy liver organoids and they were successful in discovering previously unknown genes. They found that the overexpression of C19ORF48, DTYMK, or UBE2S in HCC and C1QBP in CCs lead to ailment. They also use RNA sequencing to analyze expression patterns and found that each organoid expressed characteristic markers. HCC markers like GPC3 and AFP, as well as hepatocyte markers like ALB, TTR, APOA1, and APOE, were found to be highly expressed in HCC organoids and corresponding tissues, whereas CC and ductal markers like EPCAM, KRT19, and S100A1186, were found to be overexpressed in CC organoids, which was compatible with the features of clinical primary liver cancers. Broutier and his colleagues also demonstrated that the individual genomic profile of a patient can also get preserved during early organoid culture which is <2 months. 92% of the heterogeneity can be maintained in these organoids. In late organoid cultures, which is >4 months, 80% of the heterogeneity can be retained [66,67]. Organoids can retain the genetic, morphological, transcriptomic, and functional characteristics of the original tumors and organoid tumors can easily be compared with original tumors by examining cellular structure, tissue architecture, and protein expression patterns [68].

Single-cell RNA sequencing (scRNA-seq) has advanced greatly over the past decade. It is useful for providing new insights into the evolution and diversity of tumors as well as into the complex ecosystem in tumors. The extraordinary heterogeneity of tumors makes it q challenge for researchers to completely recapitulate the phenotype and genotype characteristics of the organ tumor and discover therapeutic interventions. Zhao et al. [42] prepared patient-derived hepatobiliary tumor organoids from seven patients and used scRNA-seq to investigate intertumoral and intratumoral heterogeneity, which exposed the intrinsic variability in the expression of transcriptional programs linked to the cell cycle, hypoxia, and epithelial state. They find proof of inherent variability of transcriptional processes along with chemo-resistance-related biological and transcriptional heterogeneity within tumor organoids. Their findings also indicated that some patients react differently towards the same medication due to resistant subpopulations with particular metabolic systems responding to different molecular markers and acquiring drug resistance. They also reported multiple genes like MET, PRKCA, PTEN, SHC1, and PIK3R1 that are related to malignancy and enrichment in cancer-related activities such as tumor proliferation, angiogenesis, and invasion that are linked to widespread treatment resistance [42].

### Potential applications of organoids in liver cancer: present and future

**Figure 2:**
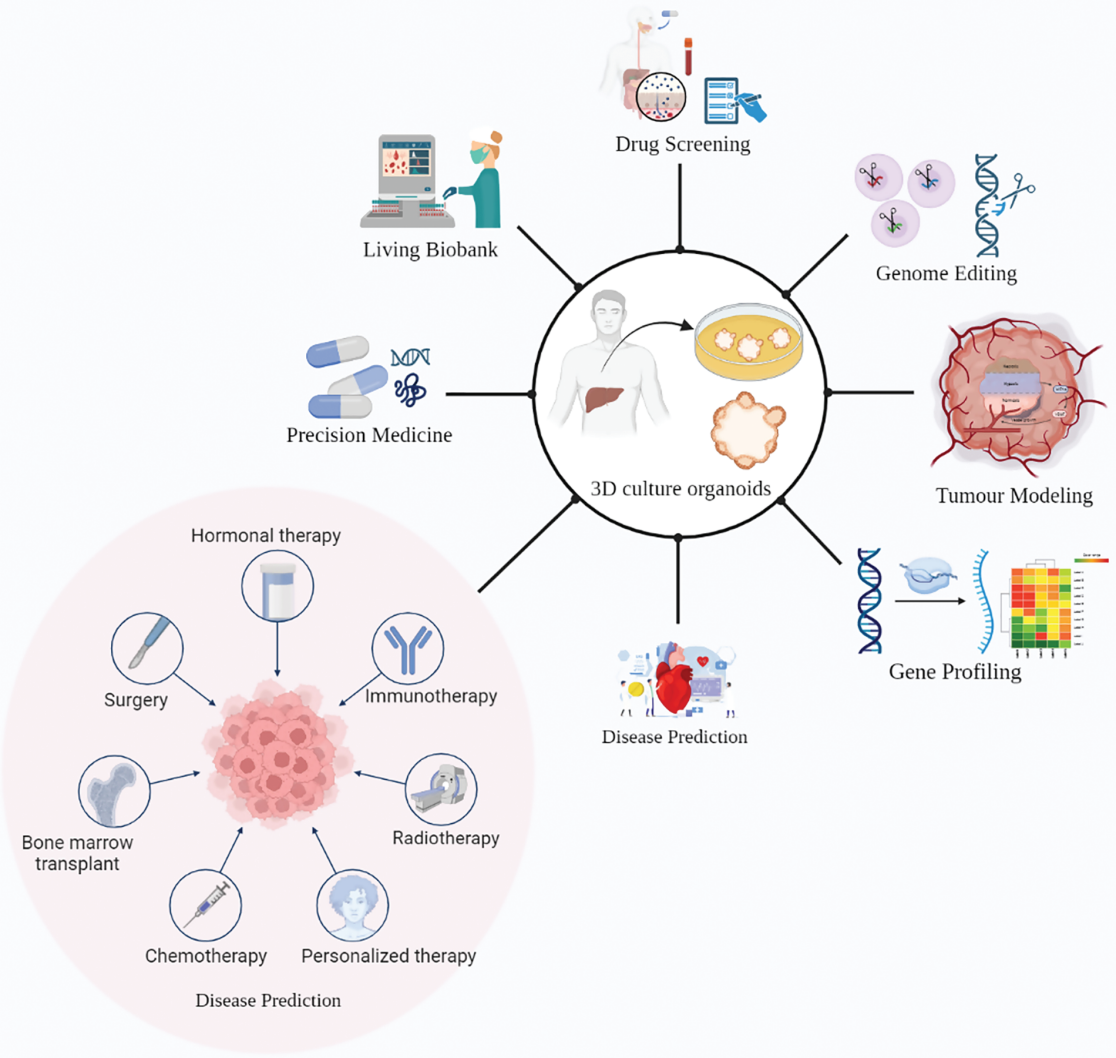
Promising ways of using liver cancer organoids in research and medical applications. These 3D cellular models can be utilized for drug screening, genome editing, precision medicine, and tumor immunotherapy. Additionally, liver cancer organoids can serve as a living biobank for disease prediction and gene profiling, as well as for studying tumor remodeling. The potential benefits of liver cancer organoids in advancing the understanding of liver cancer and improving patient outcomes are vast, highlighting the importance of continued research in this field.

Organoids have many applications when it comes to cancer research and its treatment. Organoids have the potential to remodel the original organ along with disease variants. The potential applications of tumor-derived organoids include gene profiling, drug screening, precision medicine, tumor immunotherapy, and tumor modeling (Fig. 2) [69].

During the development and progression of any cancer, many mutational mechanisms are involved, which makes it difficult to understand the source of mutational markers in tumor cells. Healthy organoids have genetic stability, which makes it possible to analyze the effects of certain mutations on the course of cancer and the mutation signature. Organoids can be used to replicate the development of and progression of tumors in certain tissues and to study their occurrence [42]. Sun et al. [70] described that hiHep organoids are important in modeling the onset of any cancer in the human body. hiHep organoids have two distinctive structural traits, Canaliculi, and the development of polarized cell-to-cell contacts, and are an attractable and transplantable system to replicate the onset of liver cancer. Additionally, they found that KRAS form normal human iCC structures, which were unable to be detected in 2D models. The Dysplastic nodule, a disease that precedes liver cancer, was modeled using hiHep organoids which demonstrated decreased hepatocyte gene expression and increased cell cycle activity, aiding oncogenic transformation. Sun and his co-workers also revealed that c-Myc and RAS were mostly responsible for the important malignant traits by using the SV40LT system which promotes the growth of hiHep cells [70]. Organoids also act as a crucial tool in hepatitis B research, revealing the molecular processes underlying hepatitis B virus (HBV) replication in a primary cell type that is scalable and appropriate for biobanking. De Crignis et al. have efficiently infected healthy donor liver organoids with HBV-infected patient serum along with recombinant virus, which leads to the generation of infectious HBV and covalently closed circular DNA in the culture supernatant. This suggests that liver organoids support whole HBV replication. This 3D cell culture infected with HBV can be employed for clinical, genomic, transcriptomic, and proteomic applications to find biomarkers for pathological conditions during HBV infection. They also identify an aberrant early cancer gene signature that may serve as a potential biomarker for HCC development and surveillance in HBV-infected patients [71]. Artegiani et al. use CRISPR/Cas9 to create human liver cancer organoids and study the underlying processes of genes implicated in the onset of tumor [69,72]. By the application of CRISPR/Cas9 in healthy cholangiocyte organoids, Artegiani et al. introduced BAP1 loss of function. In addition to other factors, the deubiquitinating enzyme BAP1 is a tumor suppressor in cholangiocarcinoma. By controlling the accessibility of chromatin, they discover that BAP1 controls the expression of junctional and cytoskeleton components, due to which some epithelial features were lost as motility increases. They also notice that reactivating BAP1 catalytic activity in the nucleus reverses these cellular and molecular alterations, which was significant. They combined four cholangiocarcinoma mutations (TP53, SMAD4, PTEN, and NF1) in human liver organoids. It was also observed that BAP1 deletion causes xenotransplantation to acquire malignant characteristics. BAP1 tumor suppressor activity thus appears to be significantly influenced by the management of epithelial identity [72]. Until the discovery of organoids, only a few models were present that accurately replicated the pathophysiology of tumor, limiting clinical trials [73,74]. According to US Food and Drug Administration, most therapeutic interventions that work effectively and safely on animals, do not work in human clinical trials [74,75]. Organoids, on the other hand, can be used for preclinical drug screening and to determine patient response to a certain medication [74]. The organoids derived from iPSCs and ASCs can completely recapitulate various stages of the disease and are thus useful in drug screening and personalized medicine [76]. To enhance prognosis and improve treatment effects in clinical studies, precision medicine is strongly advised. Liver organoids useful models for assessing the impact of experimental and clinical medication even before testing them on patients. For instance, to examine the effects of the medications metformin and I-carnitine that are prescribed to treat T2DM, a hepatic organoid system that exhibits lipid accumulation upon FFA administration has been adopted [29,77]. Similarly, Wolman disease patient liver organoids, when treated with FGF19, show decreased levels of lipid accumulation and were more likely to survive as compared to untreated organoids [21,77]. In a human 3D co-culture model of NAFLD, sorafenib was also found to reduce steatosis-induced fibrogenesis [77,78]. Inter-patient and intra-patient functional heterogeneity have been shown by Ling Li and his colleagues [79], as they tested 129 drugs on 27 primary liver cancer organoids by pan-drug screening method, producing 3483 data points on cell survival. The analyses of these drugs indicated intrinsic sensitivity to pharmaceuticals, illustrating the use of PDO lines derived from diverse malignant sites due to functional variation across patients [69,79]. One of the applications that will emerge in the future is the creation of a biobank with a large number of organoid samples. They will constitute a wide range of genetic variances which can aid in the construction of screening platforms in a global population [22]. Biobanks can be useful in swapping out conventional 2D cell lines with PDX for drug screening as researchers create biobanks of healthy and diseased human organoids through cryopreservation for biomedical uses [76]. Biobanks are also suitable for drug screening. Organoid culturing and therapy can be performed in less than 4 weeks, suggesting it to be a useful timeframe for physicians to make informed decisions. Tumor PDOs are not only useful in uncovering novel treatments but can also be used to carry out fundamental research. It is useful for understanding the mechanism of tumorigenesis and their origin and it reduces the possibility of failure of clinical trials [80]. Mo et al. established a biobank of 50 primary colon cancer tumors and paired liver metastasis [31,80]. The success rate of biobanks of liver organoids are not satisfactory. A group cultured liver tumor organoids from HCC needle biopsies and reported only a 26% success rate. Therefore it is critical to increase the success rate for producing organoids [22,28].

### Analysis of gene expression in liver cancer organoids

Gene expression analysis can help physicians to determine the prognosis of any disease. Cao et al. used Lgr5–DTR–GFP knock-in mice to investigate the presence of LGR5+ cells (GFP-coexpressing cells) in healthy and injured livers during the carcinogenesis process. Diethylnitrosamine [81] and Carbon tetrachloride (CCl_4_) were used to induce and trigger primary liver tumor formation and liver injury, respectively. Animals were monitored for 4 to 14 months. They observe that as compared to damaged liver, LGR5 expression levels vary greatly but are much higher in tumor cells. In humans, they also found that LGR5 levels are greater in tumor tissues. They also discover that the increase of LGR5 expression is more significant in HCC tumors with β-catenin mutation using data from the TCGA database, the international cancer genome consortium France (LICA-FR), and the international cancer genome consortium Japan (LICA-JP). The fact that LGR5 is a β-catenin target gene in both the gut and liver was consistent. Then, they successfully created organoid cultures of DEN-induced mice, from primary liver tumors. 89 tissue samples were obtained, of which 63 were successful in initiating the organoid process, and these organoids could last up to 5 months. After staining with CK19 and HNF4α, it was observed that these organoids display a CC or HCC-like phenotype and a FACS examination of the tumors revealed that all of them contained an LGRS5-GFP+ compartment. Upon re-culturing cells recovered from these allograft tumors as organoids, they found significant variability in morphology and CK19/HNF4 expression in the respective allograft tumor. Furthermore, the genome-wide transcriptomic analysis indicated distinctive gene expression signatures between LGR5^+^ and LGR5^-^ cells. LGR5^+^ cells had higher expression of TATA-box binding protein-associated factor 7 like (*Taf7l*), sialophorin (*Spn*), nidogen-1 (*Nid1*), SRY-box 2 (*Sox2*), alpha-1-microglobulin/bikunin precursor (*Ambp*), membrane-bound O-acyltransferase domain containing 4 (*Mboat4*), paralemmin 3 (*Palm3*), and chymase 1 (*Cma1*). This study shows that LGR5^+^ compartments are present in liver cancer and exhibit characteristics of TICs/CSCs, such exhibit an improved ability to generate tumor organoids in culture and allografts in mice as well as resistance to standard anticancer therapy [82]. The reason sorafenib caused HCC resistance was unknown [5]. Hedgehog signaling and CD44 play significant roles in TIC in HCC. By using PDOs, Dijkstra et al. [62] investigated the role of Hedgehog signaling and CD44 in sorafenib resistance and assessed the therapeutic benefits of cotreatment with sorafenib and hedgehog signaling inhibitors. They used four PDO models and their findings indicate that HCC PDOs that were CD44-positive exhibited resistance to the drug, despite sorafenib’s ability to upregulate CD44 levels. Drug testing revealed that a Hedgehog signaling inhibitor (GANT61) potently decreased HCC PDO cell survival as compared to inhibitors of Notch, Hippo, and Wnt signaling. Moreover, when sorafenib and GANT61 were introduced to CD44-positive HCC PDOs and cell lines, respectively, there was a significant synergistic decrease in cell survival and malignant characteristics *in vitro* and *in vivo*. Moreover, GANT61 inhibited sorafenib’s ability to increase CD44 and hedgehog signaling. Over the last decade, various mechanisms of resistance to targeted drugs have been identified. These include persistent targeted gene activation caused by secondary mutations, increased gene expression, or abnormal regulation of compensatory signalings, such as aberrant PI3K/AKT activation by MEK or mTOR inhibition [60].

### Gene editing using CRISPR/Cas9 in organoids

The development of the Clustered regularly interspaced short palindrome repeats (CRISPR) gene editing technology has revolutionized the field of molecular biology by enabling precise modifications to the DNA sequence of living cells. This technology has the potential to address various genetic diseases by correcting or deleting the underlying mutations. Organoids, three-dimensional structures derived from stem cells that closely resemble the *in vivo* organ, have emerged as a powerful tool for modeling human disease and drug testing. Gene editing using CRISPR/Cas9 in organoids offers an efficient and versatile approach to studying the function of genes and investigating their roles in disease pathogenesis.

**Figure 3:**
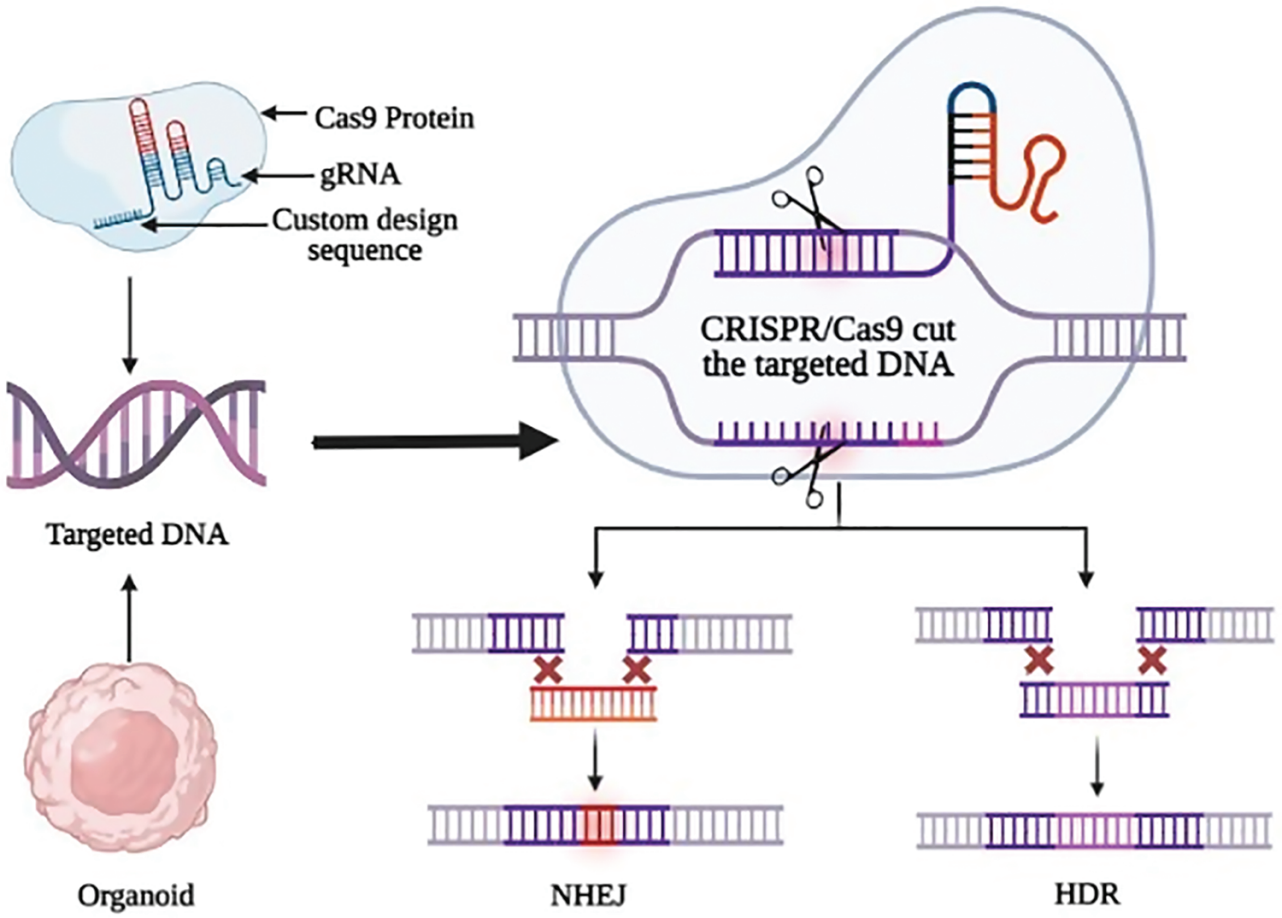
The cutting-edge technique of gene editing by CRISPR/Cas9 in organoids. The two main repair pathways, non-homologous end joining (NHEJ) and Homologous directed repair (HDR), are depicted along with their respective outcomes. Double-strand breaks can be repaired by NHEJ or HDR mechanisms. Organoids provide a useful tool for testing the efficacy of these gene editing approaches in a more complex, tissue-like environment.

The gene-editing tool has emerged as an effective tool for understanding the mechanisms underlying tumor resistance [83] as shown in Fig. 3. Cas9 can effectively control the expression of the target gene in cancer cells with the help of a single guide RNA (sgRNA), making it a powerful tool for the treatment of diseases. Liver toxicity and quick elimination of liposomal Cas9 systems pose significant obstacles to practical implementation even if liposomes via surface modification may offer cell type selectivity [84]. Effective methods for *in vitro* genome editing need to be utilized once the lines have been created to produce engineered human ASC-derived organoids. The CRISPR/Cas9 technique has greatly simplified genetic engineering [85]. Benedetta Artegiani et al. describe CRISPR/Cas9 mediated homology-independent organoid transgenesis (CRISPR-HOT), which facilitates the effective production of knock-in human organoids resembling various tissues [85]. A more advanced technology has been introduced known as CRISPR-based adenine editing since CRISPR/Cas9-mediated homology-dependent repair can cause potentially hazardous off-target double-stranded breaks. This technique allows precise enzymatic conversion of A-T base pairs into G-C base pairs, allowing for on-target base editing that aids in the restoration of mutation-affected function [86,87]. CRISPR-based genetic mutations reveal vital information about cancer onset. To mimic the early stages of liver cancer Sun et al. used genetically modified hepatocyte organoids and reported that the malignant HCC was caused by the expression c-Myc in contrast to RAS^G12V^, YAP^5SA^, IDH2^R172K^, and PTPN^3A90P^ mutations in organoids that developed morphological changes similar to intrahepatic cholangiocarcinoma [66,70]. It may also be used to investigate the biological role of disease risk alleles highlighted by genome-wide association studies, which cannot identify the underlying target genes and cell types [77,88]. In patient-derived hepatic organoids, CRISPR-Cas9 and base editing have also been used to restore a disease-causing mutation to the wild-type state to reverse Alagille syndrome and Wilson disease phenotypes, offering an effective treatment strategy for a disorder of the liver [77,89].

Despite these advancements, the current approach of generating liver organoids has several limitations that can impede their effectiveness in recapitulating the complexity of the liver tissue. One of the main challenges is the lack of precise genetic manipulation capabilities to study specific gene functions and disease mechanisms [90]. This is where the CRISPR/Cas9 system comes into play, offering a revolutionary tool for gene editing in liver organoids. By utilizing CRISPR/Cas9, researchers can precisely modify the genome of liver organoids, allowing for the investigation of gene functions, disease modeling, and the identification of potential therapeutic targets [20]. Studies have demonstrated the successful application of CRISPR/Cas9 in liver organoids, enabling the correction of disease-causing mutations, the introduction of specific genetic modifications, and the elucidation of gene regulatory networks [91–93]. Therefore, the integration of the CRISPR/Cas9 system in liver organoid research has the potential to overcome the current limitations and pave the way for more accurate and robust investigations into liver biology and disease mechanisms.

In the future, the CRISPR/Cas9 system can be used for targeted gene editing in liver organoids to study disease mechanisms and develop personalized treatments for liver diseases. A study by Song et al. [94] demonstrated the use of CRISPR/Cas9 to correct a disease-causing mutation in liver organoids derived from a patient with α1-antitrypsin deficiency [94]. Another study by Lim et al. showed the potential of CRISPR/Cas9 for correcting mutations in liver organoids and generating personalized models for drug testing [95]. These promising results of using CRISPR/Cas9 in liver organoids have shown potential for future use in precision medicine.

### Challenges and prospects of organoids in liver cancer research

As compared to the monolayer 2D model, organoids bear a great resemblance to the tissue of the origin along with the recapitulation of heterogeneity. However, some challenges need to be improved upon [91]. One of the obstacles in organoid research is the use of Matrigel, because it is derived from the mouse sarcoma cell and most of its components are unknown. This increases variability and causes poor reproducibility in organoid research. Apart from making it more difficult to evaluate the metabolic activities of adipose and liver organoids, the incorporation of Matrigel in the culture system also makes it more difficult for organoid passaging, manipulating their genetic makeup, performing genome-wide screening, and conducting high throughput drug screening. Future studies of metabolic diseases using organoids will be more affordable, reproducible, and translatable because of the development of synthetic and adaptable scaffolds [77]. *In vitro*, organoids cannot accurately mimic all the cell types and maturation of the organ of origin, as they do not retain dynamic physiological processes, and most often, a majority of PDOs also lack stromal cells and signaling that promotes organogenesis. This makes it difficult to predict any clinical utility as they are unable to accurately recreate the TME which consists of fibroblasts, endothelial cells, immune cells, and the ECM. The survival rate of these models is quite short and over the course of 1–2 months, the survival of fibroblast and immune cells starts to decline. Additionally, organoids lack vascular components to support appropriate nutrition absorption which causes the size of organoid models to be restricted. Despite the presence of new techniques like tumor-on-a-chip and vascularized organoids, vascularization remains a challenge. As a consequence, organoids are unable to accurately recapitulate the pathogenic and developmental traits of primary organs. The tissue-specific micro-physiology of human organoids is also not very extensive. The production of PDOs is quite expensive and it cannot be produced on a large scale due to its short life span [69]. Despite gene editing being a game-changing technology in organoid research, some challenges are still present, like off-target effects, mutagenesis, tumorigenesis, etc. Off-target effects can cause random integration, which may lead to insertional mutagenesis and eventually lead to cancer in transplanted cells [96]. The combination of immune, stromal, and cancer cells to produce organoids is still under investigation [97].

## Conclusion and Prospects

3D Organoids have a huge impact on the field of cancer treatment and research. Despite the vast number of advantages, organoids have some limitations that need to be overcome via proper standardization of protocols or otherwise. To study the evolution of carcinogenesis, a stepwise characterization of various mutations is needed. Further developments and analyses are required to allow accurate predictions in genetic and transcriptomic pathways and considerable progress in genome editing may facilitate this. In the future, such techniques will be very helpful in achieving control over organoid research to investigate various cancer subtypes but this may be expensive and time taking. Efforts should be made to lower the costs of organoid research so that practitioners can apply this technique in healthcare sectors on a large scale, bringing about breakthrough developments in cancer research and therapy.

## Data Availability

Not applicable.
